# Sex-specific associations of low birth weight with adult-onset diabetes and measures of glucose homeostasis: Brazilian Longitudinal Study of Adult Health

**DOI:** 10.1038/srep37032

**Published:** 2016-11-15

**Authors:** James Yarmolinsky, Noel T Mueller, Bruce B Duncan, Dóra Chor, Isabela M Bensenor, Rosane H Griep, Lawrence J Appel, Sandhi M Barreto, Maria Inês Schmidt

**Affiliations:** 1 Postgraduate Program in Epidemiology, School of Medicine, Federal University of Rio Grande do Sul, Porto Alegre, RS, Brazil; 2 Department of Epidemiology, Johns Hopkins Bloomberg School of Public Health, Baltimore, MD, USA; 3 Welch Center for Prevention, Epidemiology and Clinical Research, Johns Hopkins University, Baltimore, MD, USA; 4 Department of Epidemiology, University of North Carolina, Chapel Hill, NC, USA; 5 National School of Public Health, Fundação Oswaldo Cruz, Rio de Janeiro, RJ, Brazil; 6 Department of Clinical Medicine, Faculty of Medicine, University of São Paulo, São Paulo, SP, Brazil; 7 Center for Clinical and Epidemiological Research, University Hospital, University of São Paulo, São Paulo, SP, Brazil; 8 Laboratory of Health and Environment Education, Oswaldo Cruz Institute, Oswaldo Cruz Foundation, Rio de Janeiro, RJ, Brazil; 9 Faculty of Medicine, Federal University of Minas Gerais, Belo Horizonte, MG, Brazil

## Abstract

Emerging evidence suggests sex differences in the early origins of adult metabolic disease, but this has been little investigated in developing countries. We investigated sex-specific associations between low birth weight (LBW; <2.5 kg) and adult-onset diabetes in 12,525 participants from the Brazilian Longitudinal Study of Adult Health (ELSA-Brasil). Diabetes was defined by self-reported information and laboratory measurements. In confounder-adjusted analyses, LBW (vs. 2.5–4 kg) was associated with higher prevalence of diabetes in women (Prevalence Ratio (PR) 1.54, 95% CI: 1.32–1.79), not in men (PR 1.06, 95% CI: 0.91–1.25; *P*_heterogeneity_ = 0.003). The association was stronger among participants with maternal diabetes (PR 1.60, 95% CI: 1.35–1.91), than those without (PR 1.15, 95% CI: 0.99–1.32; *P*_heterogeneity_ = 0.03). When jointly stratified by sex and maternal diabetes, the association was observed for women with (PR 1.77, 95% CI: 1.37–2.29) and without (PR 1.45, 95% CI: 1.20–1.75) maternal diabetes. In contrast, in men, LBW was associated with diabetes in participants with maternal diabetes (PR 1.45, 95% CI: 1.15–1.83), but not in those without (PR 0.92, 95% CI: 0.74–1.14). These sex-specific findings extended to continuous measures of glucose homeostasis. LBW was associated with higher diabetes prevalence in Brazilian women, and in men with maternal diabetes, suggesting sex-specific intrauterine effects on adult metabolic health.

A large Danish cohort study recently provided intriguing evidence that the well-established association between low birth weight and adult diabetes varies by sex^1^, corroborating evidence from famine studies^2^,^3^,^4^,^5^,^6^,^7^and mice models that the metabolic response to an adverse intrauterine environment is sex dependent^8^,^9^,^10^,^11^. An important Bradford Hill criterion^12^ for inferring whether sex differences in the early origins of disease hypothesis are causal is to examine if the sex-specific association between low birth weight and diabetes is *reproducible* in less economically prosperous settings, where the etiology of both low birth weight and diabetes may differ.

In addition to low birth weight, maternal diabetes, a rapidly increasing problem globally^13^,^14^, may lead to epigenetic changes during fetal development that increase future risk of adult diabetes^15^,^16^,^17^,^18^,^19^,^20^. To the best of our knowledge, no studies have investigated whether maternal diabetes modifies the association of low birth weight and adult-onset diabetes.

Thus, the aim of the current study was to examine sex-specific associations of low birth weight with adult diabetes and measures of glucose homeostasis in a large multi-ethnic contemporary cohort of middle-aged and elderly Brazilians, who were born an era of chronic food insecurity but who now live in an era of easy food access. Moreover, we will capitalize on our large cohort size to explore whether having a mother with diabetes, a marker of intrauterine metabolic dysregulation, modifies the birth weight-diabetes association in a sex-specific manner.

## Methods

### Study design and population

The Brazilian Longitudinal Study of Adult Health (in Portuguese, Estudo Longitudinal de Saude do Adulto or ELSA-Brasil) is a cohort study of 15,105 volunteer employees, aged 35 to 74 years at baseline (2008–2010), from universities or research institutions located in six cities in three different regions of Brazil. The details of the study, including design, eligibility criteria, sources and methods of recruitment, and measurements obtained, have been described in detail elsewhere^21^,^22^. ELSA-Brasil was approved by the Ethics Committees of the Hospital de Clínicas de Porto Alegre (06-194), Hospital Universitário da Universidade de São Paulo (669/06), Fundação Oswaldo Cruz (343/06), Universidade Federal de Minas Gerais (186/06), Universidade Federal da Bahia (027-06) and Universidade Federal do Espírito Santo (041/06). Informed consent was obtained from all participants involved in this analysis. All methods were performed in accordance with the relevant guidelines and regulations.

### Exposure and covariate assessment

We applied standardized questionnaires and measurements^8^. We asked participants to recall their weight at birth as “less than 2.5 kg”, “2.5 to 4 kg”, “greater than 4 kg”, or as “I don’t know”. These first three categories were defined as low birth weight, normal birth weight, and high birth weight, respectively. Participants were also asked if they could provide their specific birth weight in kg.

Age at menarche was assessed by asking women the open-ended question, “At what age did you menstruate for the first time ?”. History of paternal diabetes was ascertained by asking participants “Was your father diagnosed with diabetes?” and history of maternal diabetes as “Was your mother diagnosed with diabetes?”. Participants who answered, “I don’t know” to either of these parental history of diabetes questions were categorized as “no” for each respective covariate in this analysis. Maternal educational attainment was ascertained by asking participants “What was the highest level of education that your mother completed?”. Body mass index (BMI) was calculated as measured weight (kg) divided by measured height squared (m^2^).

### Outcome ascertainment

Self-reported diabetes and use of diabetic medication were assessed by questionnaire. We drew fasting blood samples and then administered a standard 75 g oral glucose tolerance test to participants without known diabetes. Glucose was measured by the hexokinase method (ADVIA Chemistry; Siemens, Deerfield, Illinois), glycated hemoglobin (HbA_1c_) using a high-pressure liquid chromatography (Bio-Rad Laboratories, Hercules, California), a National Glycohemoglobin Standardization Program (NGSP) certified method, and insulin with an immunoenzymatic assay (ELISA) (Siemens).

Diabetes was classified when a participant responded “yes” to either of the following questions: “Have you been previously told by a physician that you had diabetes (sugar in the blood)?” or “Have you used medication for diabetes in the past 2 weeks?”. Those who responded “no” to both questions, were classified as having diabetes when reaching the threshold for fasting plasma glucose (≥7.0 mmol/L [≥126 mg/dL]), 2-hour postload plasma glucose (≥11.1 mmol/L [≥200 mg/dL]), or HbA_1c_ (≥6.5%)^23^,^24^.

Homeostatic model assessment of insulin resistance (HOMA-IR) was defined as the product of fasting glucose (mmol/L) and insulin levels (μIU/mL) divided by 22.5; homeostatic model assessment of β cell function (HOMA-β) as the product of 20 and fasting insulin (μIU/mL) divided by fasting glucose (mmol/L) minus 3.5^25^. Whole body insulin sensitivity was assessed using the insulin sensitivity index (ISI-composite) proposed by DeFronzo and Matsuda applied to the fasting and 2 hour values^26^.

### Statistical analyses

For the current investigation we used data collected at baseline. We excluded participants with missing data for the following variables: self-recalled birth weight (n = 2228), race/skin color (n = 184), maternal education level (n = 366), BMI (n = 6), and laboratory measurements (n = 17). Some participants had missing data for two or more variables. Our main analyses were performed in 12,525 participants (82.9% of ELSA-Brasil).

Poisson regression with robust variance was performed to generate prevalence ratios (PRs) and 95% confidence intervals (CIs) for the association of categories of birth weight with diabetes. In Model 1, we present a model adjusted for age and study center. In Model 2, we additionally include risk factors for low birth weight and diabetes that were unlikely to lie on the causal pathway between birth weight and adult diabetes (race/color, maternal education, father diagnosed with diabetes). In Model 3, we additionally add measured BMI obtained at the ELSA site to investigate its potential mediation role in the association.

Analysis of covariance (ANCOVA) was performed to assess the association between category of birth weight and markers of glucose and insulin homeostasis as continuous variables. Participants with previously known diabetes or missing laboratory values were excluded from these analyses (n = 1,361). All continuous markers of glucose and insulin homeostasis were log-transformed then converted to geometric means.

We performed sensitivity analyses including only participants who reported an exact birth weight consistent with their categorically reported birth weight, and sensitivity analyses excluding participants with self-reported preterm birth.

We first tested for multiplicative interaction by sex. We then tested for interactions by race/skin color (white vs. non-white), year of birth (1970 cut-point), age at menarche (≤12 years, >12 years), BMI at the baseline examination (<25 kg/m^2^, ≥25 and <30 kg/m^2^, ≥ 30 kg/m^2^), maternal history of diabetes (yes vs. no), and paternal history of diabetes (yes vs. no). If a one-way interaction for these variables was found, we proceeded to test for a sex-specific interaction. All statistical tests were two-sided and significance was defined at *P* < 0.05. Statistical analyses were performed with SAS 9.4 (SAS Institute, Inc., Cary, North Carolina).

## Results

Participants in our sample were, on average, 51.3 (SD = 8.8) years of age (range: 34–75 y). As seen in Table 1, the majority (n = 10,542 or 84%) of participants reported their birth weight as between 2.5 and 4 kg. Another 1,043 (around 8% of the total) reported their birth weight as <2.5 kg, and 940 (around 8% of the total) reported their birth weight as >4 kg. Generally, compared to participants with high birth weight, those with low birth weight were more likely to be female, black or “pardo”, and have a mother with low educational attainment. There were 2,310 cases of diabetes.

Testing for heterogeneity in the association of birth weight with diabetes identified evidence for interaction by sex (*P*_heterogeneity_ = 0.01). In multivariable-adjusted confounder models (Model 2) stratified by sex, we found an association between low birth weight and diabetes in women (PR 1.54, 95% CI 1.32–1.79), but not in men (PR 1.06, 95% CI 0.91–1.25; *P*_heterogeneity_ = 0.003) (Table 2). In women, this association was maintained in a model further adjusted for BMI at baseline (Model 3). We also found evidence for interaction of the association of birth weight with diabetes by maternal diabetes (*P*_heterogeneity_ = 0.046). In multivariable analyses stratified by maternal diabetes, there was a stronger association between low birth weight and diabetes among participants whose mother was diagnosed with diabetes (PR 1.60, 95% CI 1.35–1.91), compared to participants whose mother was not (PR 1.15, 95% CI 0.99–1.32; *P*_heterogeneity_ = 0.03). Further adjustment for BMI did not materially change these associations. No difference was found in the association of high birth weight with diabetes by maternal diabetes status.

When we tested for a two-way interaction of the association of birth weight and diabetes with both sex and maternal diabetes status, we found evidence for a maternal diabetes-specific association of birth weight with diabetes among men (*P*_heterogeneity_ = 0.03), but similar associations of birth weight with diabetes among women regardless of maternal diabetes status (*P*_heterogeneity_ = 0.57). Specifically, among men, in multivariable-adjusted confounder analyses, low birth weight was associated with diabetes in participants with a maternal history of diabetes (PR 1.45, 95% CI 1.15–1.83), but not in those without maternal diabetes (PR 0.92, 95% CI 0.74–1.14) (Table 3). In contrast, among women, similar associations of low birth weight and diabetes were reported between those with maternal diabetes (PR 1.77, 95% 1.37–2.29) and those without maternal diabetes (PR 1.45, 95% CI 1.20–1.75). Further adjustment for BMI did not materially change these associations.

We then examined the association of birth weight with continuous glycemic measures in women and men separately. In women, we found inverse associations of birth weight with fasting and 2-hour post load glucose and positive linear associations with HOMA-β among those with and without a maternal history of diabetes (Table 4). We also found an inverse association of birth weight with HOMA-IR and a positive linear association with ISI-composite, solely among participants without maternal diabetes. In men, we found evidence for an inverted “U-shaped” association of birth weight with ISI-composite and an inverse association with 2-hour postload glucose, solely among men without maternal diabetes (Table 5).

Our results did not appreciably change in sensitivity analyses when we included only those participants who were able to recall an exact birth weight consistent with their categorical recall of birth weight, or when we removed participants with self-reported pre-term births (Supplementary Tables S1 and S2).

Overall, high birth weight was associated with a reduced adjusted prevalence of adult-onset diabetes (PR 0.80, 95% CI 0.75–0.87). As seen in Tables 2 and 3, the strength of this association did not differ across analyses stratified by sex and maternal diabetes status.

Finally, we did not find evidence for interactions of birth weight with race/skin color (*P* = 0.09), year of birth (P = 0.08), age at menarche (*P* = 0.41), BMI at the baseline examination (*P* = 0.67), or paternal diabetes (*P* = 0.81).

## Discussion

In this analysis of 12,525 middle-aged and elderly Brazilians, we found sex-specific associations with low birth weight and prevalence of adult-onset diabetes and adult measures of glucose homeostasis (fasting and post-load hyperglycemia, insulin resistance, and β cell dysfunction). Overall, the association between low birth weight and adult-onset diabetes was stronger among those with a maternal history of diabetes. When we stratified results by both sex and maternal diabetes status, low birth weight was associated with elevated diabetes prevalence in women regardless of maternal diabetes status, while in men only when a maternal history of diabetes was present.

Our sex-specific findings are consistent with a recent report investigating 223,099 Danish adults in which women with low birth weight (2–2.75 kg) compared to women with mid-to-high birth weight (3.75–4.75 kg) had greater risk of T2D in adulthood (HR [Hazard Ratio] 1.46, 95% CI 1.34–1.59). The association was considerably weaker in men with low birthweight (HR 1.20, 95% CI 1.12–1.30)^1^. Other studies that have investigated this topic in LMICs (low- and middle-income countries)^27^,^28^,^29^,^30^,^31^ have been conducted in cohorts that were younger and considerably smaller in sample size than ours, which may explain why none of these studies examined effect modification by sex on the association between low birth weight and diabetes, and only one of these studies^27^ evaluated and found evidence of effect modification by sex on the association between low birth weight and an early marker of impaired glucose homeostasis (i.e., elevated fasting insulin).

While sex differences have been infrequently reported for the relationship between low birth weight and diabetes, there have been reports of effect modification by sex in the association of other measures of intrauterine nutrition and adult cardiometabolic disease. For example, studies of middle-aged adults conceived during the Dutch Famine of 1944–1945, the Nigerian Biafran Famine, the Great Depression in Reykjavik (1930–1934), and the Great Chinese Famine have reported intrauterine food scarcity is more strongly associated with elevated measures of adiposity^2^, lipids^5^, fasting plasma glucose^4^, hypertension^6^, the metabolic syndrome^7^, and diabetes in women than men^32^.

The sex-specific findings in our study and the famine studies may reflect greater survival of females exposed to under nutrition *in utero*. The Great Chinese Famine, produced approximately a 7% decrease in the male:female ratio of the affected birth cohort when measured 40 years later^33^. Higher mortality rates in men than in women following *in utero* exposure to the Dutch Potato Famine of 1846–1847 and the Korean War have also been reported^34^,^35^ and several studies report greater male than female deaths among low birth weight infants^36^,^37^.

The sex-specific associations between low birth weight and diabetes may also reflect sexually dimorphic metabolic responses to intrauterine environs that persist throughout life. Mouse studies suggest that fetal under nutrition may have more impact on the development of hyperglycemia, insulin resistance, and a diminished insulin secretory response in females than males^8^. Additionally, mitochondrial oxidative phosphorylation, a key determinant of energy homeostasis and contributor to insulin resistance and diabetes, was enhanced in female piglets, but not in male piglets, following a maternal low protein diet^38^,^39^. Elevated maternal testosterone during pregnancy associates with low birth weight in offspring^40^ and maternal and fetal testosterone levels have been shown to highly correlate^41^. As elevated testosterone levels are associated with higher risk of T2D (type 2 diabetes) in women, but lower T2D risk in men^42^, it is possible that mechanisms related to sex-steroid hormones underlie the observed sex differences. Birth weight is positively correlated with serum leptin levels in both sexes^43^, but elevated leptin is associated with risk for adult diabetes in a sex-specific manner^44^, suggesting leptin/leptin resistance as another pathway that may explain our sex-specific findings.

Our sex-specific findings—overall and those related to a history of maternal diabetes—may also be due to sex differences in epigenetic programming caused by intrauterine environmental insults. Sex-specific DNA methylation patterns have been observed in the cord blood of neonates following intrauterine growth restriction^45^, and in middle-aged adults following prenatal exposure to the Dutch Famine of 1944–1945^46^. Additionally, several studies have demonstrated alteration of DNA methylation patterns in offspring of diabetic mothers^15^,^20^, but to the best of our knowledge, we are the first to show that maternal diabetes may modify the association between intrauterine growth restriction and adult-onset diabetes in a sex-specific manner. DNA methylation of loci associated with insulin resistance and type 2 diabetes in infants born to mothers with gestational diabetes and in infants born with growth restriction demonstrates large changes in methylation but surprisingly little overlap in specific methylated loci by sex^47^. The fact that we only found that low birth weight men with maternal history of diabetes have increased risk of diabetes suggest factors leading to low birth weight in men other than maternal diabetes are not sufficient to confer diabetes risk. Future investigation is needed to determine if this sex-specific interaction of maternal diabetes and birth weight on adult-onset of diabetes is reproducible.

Lastly, consistent with some^48^, but not all^49^, previous studies we found that high birth weight was associated with a reduced adjusted prevalence of adult-onset diabetes. The strength of this association did not differ across analyses stratified by sex and maternal diabetes status.

Our study has several strengths. First, it is the largest investigation of birth weight in relation to adult-onset diabetes risk in a LMIC context to date. The relatively large size of our cohort has afforded the ability to explore sex-specific interactions that previous studies from LMICs have not had the power to examine. Second, the relatively advanced age of the ELSA-Brasil sample has allowed us to extend the previous studies from LMICs – mainly birth cohorts – that typically examined health outcomes among young adults. Third, we believe our results have wide generalizability. The majority of the world´s population is currently undergoing a rapid nutritional transition. ELSA participants, different from those in high-income countries, underwent this rapid transition. They were born between 1930 and 1970, and thus lived through the mid-1900s during which food insecurity was common, as well as through the more recent period in which diabetes has been highly prevalent. Finally, the use of a standard 75 g oral glucose tolerance test for those without previously diagnosed diabetes and measurements of glycated hemoglobin for all participants allowed us to provide a broader and more sensitive assessment of diabetes than all previous studies from LMICs on this topic.

Some limitations of our study deserve mention. First, birth weight data were based on self-report. Previous studies have shown moderate correlations between estimated and recorded birth weight (r = 0.64–0.83)^50^,^51^,^52^. We expect but cannot confirm that misclassification of birth weight in our study of older adults, if present, would be non-differential with respect to diabetes, and therefore likely underestimate the strength of association reported. Second, it is important to note that a maternal history of diabetes implies a diagnosis made at any time in the maternal life course. Nevertheless, given the natural history of T2D, it is likely that at the time of pregnancy some degree of glucose dysregulation would have been present, and a considerable proportion of these women may have reached a degree of hyperglycemia compatible with gestational diabetes. Third, we did not have information to characterize the causes of low birth weight in our participants, and thus we cannot infer what causes of low birth weight are driving the birth weight-T2D association. Finally, although we had a priori hypotheses about sex-specific associations, we cannot rule out that one or more of our findings were due to chance, as many statistical tests were performed.

In sum, we observed a sex-specific association between low birth weight and adult-onset diabetes in a contemporary cohort of Brazilian adults. These findings, consistent with those from epidemiological studies and experimental animal studies, suggest the fetal origins of diabetes hypothesis could be extended to include sex-specific environmental causes. The relevance and complexity of these sex differences in today´s environment is an exciting area for future epidemiological and basic science research.

## Additional Information

**How to cite this article**: Yarmolinsky, J. *et al.* Sex-specific associations of low birth weight with adult-onset diabetes and measures of glucose homeostasis: Brazilian Longitudinal Study of Adult Health. *Sci. Rep.* **6**, 37032; doi: 10.1038/srep37032 (2016).

**Publisher’s note**: Springer Nature remains neutral with regard to jurisdictional claims in published maps and institutional affiliations.

## Supplementary Material

Supplementary Information

## Figures and Tables

**Table 1:** Descriptive data of ELSA-Brasil participants (N = 12525) at baseline (2008–2010) according to birth weight category.

Birth weight categories				
	<2.5 kg	≥2.5, ≤4 kg	>4 kg	*P*-value
Total	1,043 (8.3)	10,542 (84.2)	940 (7.5)	
Female	598 (57.3)	5890 (55.9)	400 (42.6)	<0.0001
Age, years	51.4 ± 8.7	51.3 ± 8.9	50.7 ± (8.7)	0.12
Race/color				
Black	178 (17.1)	1578 (15.0)	128 (13.6)	<0.0001
Brown “Pardo”	320 (30.7)	2849 (27.0)	233 (24.8)	
White	486 (46.6)	5789 (54.9)	558 (59.4)	
Other^a^	59 (5.7)	326 (3.1)	21 (2.2)	
Diabetes prevalence	255 (24.4)	1894 (18.0)	161 (17.1)	<0.0001
BMI at baseline, kg/m^2^	26.7 ± 4.9	27.0 ± 4.7	28.3 ± 5.3	<0.0001
Low maternal educational attainment^b^	658 (63.1)	5693 (54.0)	478 (50.8)	<0.0001
Mother diagnosed with diabetes	217 (20.8)	2033 (19.3)	252 (26.8)	<0.0001
Father diagnosed with diabetes	127 (12.2)	1420 (13.5)	138 (14.7)	0.10

Values are means and standard deviations for continuous variables and frequencies and percentages for categorical variables. Abbreviations: BMI, body mass index (kg/m^2^).

^a^“Other”: those reporting their race/skin color as “Asian” or “Indigenous.

^b^Less than complete elementary school. *P*-value represents the test for an overall association of the different categories of estimated birth weight with each respective covariate.

**Table 2:** Multivariable adjusted prevalence ratios (and 95% confidence intervals) for the association of birth weight categories with adult-onset diabetes, stratified by sex and maternal diabetes: ELSA-Brasil (N = 12525).

Birth weight categories				
	<2.5 kg	≥2.5, ≤4 kg	>4 kg	*P*-value
Men				
*n*	445	4652	540	
Cases	116	1028	104	
Model 1	1.15 (0.97–1.35)	1 (reference)	0.91 (0.77–1.09)	0.15
Model 2	1.06 (0.91–1.25)	1 (reference)	0.89 (0.75–1.06)	0.27
Model 3^†^	1.10 (0.94–1.30)	1 (reference)	0.80 (0.67–0.96)	0.01
**Women**				
*n*	598	5890	400	
Cases	139	866	57	
Model 1	1.63 (1.40–1.90)	1 (reference)	1.00 (0.78–1.27)	<0.0001
Model 2	1.54 (1.32–1.79)	1 (reference)	0.95 (0.75–1.21)	<0.0001
Model 3^†^	1.53 (1.32–1.78)	1 (reference)	0.81 (0.65–1.02)	<0.0001
**Maternal diabetes**				
*n*	217	2033	252	
Cases	91	504	57	
Model 1	1.66 (1.40–1.96)	1 (reference)	0.91 (0.72–1.15)	<0.0001
Model 2^‡^	1.60 (1.35–1.91)	1 (reference)	0.90 (0.71–1.13)	<0.0001
Model 3	1.61 (1.35–1.91)	1 (reference)	0.81 (0.65–1.02)	<0.0001
**No maternal diabetes**				
*n*	826	8509	688	
Cases	164	1390	104	
Model 1	1.23 (1.07–1.41)	1 (reference)	0.98 (0.82–1.16)	0.03
Model 2^‡^	1.15 (0.99–1.32)	1 (reference)	0.93 (0.78–1.11)	0.13
Model 3	1.18 (1.02–1.36)	1 (reference)	0.81 (0.68–0.97)	0.003

Model 1: adjusted for age, study center; Model 2: + race/color, maternal education, father diagnosed with diabetes; Model 3: + BMI at baseline.

^†^Further adjusted for mother diagnosed with diabetes.

^‡^Further adjusted for sex. *P*-value represents the test for an overall association of the different categories of estimated birth weight with diabetes. *P*-values were obtained from chi-square tests which measured the divergence of the observed frequencies of adult-onset diabetes across birth weight categories from the values that would be expected under the null hypothesis of no association.

**Table 3:** Multivariable adjusted prevalence ratios (and 95% confidence intervals) for the association of birth weight categories with adult-onset diabetes, stratified jointly by sex and maternal diabetes: ELSA-Brasil (N = 12525).

Birth weight categories				
	<2.5 kg	≥2.5, ≤4 kg	>4 kg	*P*-value
**Men whose mothers had history of diabetes**				
*n*	91	887	130	
Cases	43	265	34	
Model 1	1.53 (1.21–1.92)	1 (reference)	0.85 (0.63–1.15)	0.004
Model 2	1.45 (1.15–1.83)	1 (reference)	0.86 (0.64–1.15)	0.01
Model 3	1.54 (1.23–1.93)	1 (reference)	0.81 (0.60–1.10)	0.002
**Men whose mothers did not have history of diabetes**				
*n*	354	3765	410	
Cases	73	763	70	
Model 1	0.98 (0.80–1.21)	1 (reference)	0.91 (0.73–1.12)	0.64
Model 2	0.92 (0.74–1.14)	1 (reference)	0.91 (0.74–1.13)	0.53
Model 3	0.94 (0.75–1.17)	1 (reference)	0.80 (0.64–1.00)	0.09
**Women whose mothers had history of diabetes**				
*n*	126	1146	122	
Cases	48	239	23	
Model 1	1.78 (1.39–2.29)	1 (reference)	0.94 (0.65–1.37)	0.001
Model 2	1.77 (1.37–2.29)	1 (reference)	0.98 (0.68–1.41)	0.002
Model 3	1.66 (1.28–2.17)	1 (reference)	0.82 (0.57–1.17)	0.002
**Women whose mothers did not have history of maternal diabetes**				
*n*	472	4744	278	
Cases	91	627	34	
Model 1	1.55 (1.28–1.87)	1 (reference)	0.93 (0.68–1.28)	0.0008
Model 2	1.45 (1.20–1.75)	1 (reference)	0.93 (0.68–1.26)	0.003
Model 3	1.50 (1.26–1.79)	1 (reference)	0.80 (0.60–1.08)	0.0002

Model 1: adjusted for age, study center; Model 2: + race/color, maternal education, father diagnosed with diabetes; Model 3: + BMI at baseline. *P*-value represents the test for an overall association of the different categories of estimated birth weight with diabetes. *P*-values were obtained from chi-square tests which measured the divergence of the observed frequencies of adult-onset diabetes across birth weight categories from the values that would be expected under the null hypothesis of no association.

**Table 4:** Multivariable adjusted mean (SE) of measures of glucose homeostasis and insulin resistance and secretion in women across categories of birth weight, stratified by maternal diabetes status: ELSA-Brasil (2008–2010).

Birth weight categories				
	<2.5 kg	≥2.5, ≤4 kg	>4 kg	*P*-value
**Women whose mothers had history of diabetes**^**1**^				
**Fasting glucose (mmol/l)**				
Model 1	5.97 (0.08)	5.82 (0.04)	5.75 (0.08)	0.08
Model 2	5.97 (0.08)	5.85 (0.04)	5.71 (0.08)	0.03
**2 h postload glucose (mmol/l)**				
Model 1	7.73 (0.25)	7.20 (0.12)	6.96 (0.21)	0.02
Model 2	7.72 (0.24)	7.30 (0.12)	6.82 (0.20)	0.004
**HOMA-IR**				
Model 1	1.43 (0.15)	1.21 (0.07)	1.45 (0.14)	0.03
Model 2	1.42 (0.13)	1.29 (0.06)	1.31 (0.11)	0.56
**HOMA-β**^**†**^				
Model 1	45.63 (1.94)	48.85 (1.12)	52.14 (2.10)	0.04
Model 2	45.69 (1.93)	48.70 (1.11)	52.71 (12.12)	0.02
**ISI-composite**				
Model 1	4.87 (0.45)	5.85 (0.29)	5.40 (0.47)	0.08
Model 2	4.90 (0.40)	5.50 (0.24)	5.91 (0.46)	0.17
**Women whose mothers did not have history of diabetes**^**2**^				
**Fasting glucose (mmol/l)**				
Model 1	5.86 (0.03)	5.77 (0.02)	5.77 (0.04)	0.01
Model 2	5.87 (0.03)	5.76 (0.02)	5.73 (0.04)	0.0004
**2 h postload glucose (mmol/l)**				
Model 1	7.13 (0.10)	6.92 (0.05)	6.79 (0.12)	0.03
Model 2	7.17 (0.10)	6.90 (0.05)	6.67 (0.11)	0.0007
**HOMA-IR**				
Model 1	1.47 (0.07)	1.35 (0.03)	1.38 (0.08)	0.16
Model 2	1.52 (0.07)	1.34 (0.03)	1.23 (0.07)	0.001
**HOMA-β**^**†**^				
Model 1	44.76 (0.80)	46.67 (0.44)	46.95 (1.04)	0.04
Model 2	44.44 (0.79)	46.70 (0.44)	47.53 (1.04)	0.007
**ISI-composite**				
Model 1	5.06 (0.22)	5.49 (0.13)	5.79 (0.31)	0.07
Model 2	4.92 (0.19)	5.55 (0.11)	6.35 (0.30)	<0001

Model 1: adjusted for age, study center, maternal education, father diagnosed with diabetes; Model 2: + BMI at baseline.

^†^Further adjusted for HOMA-IR. *P*-value represents the test for an overall association of the different categories of estimated birth weight with each respective outcome.

^1^N = 1180.

^2^N = 4994.

**Table 5:** Multivariable adjusted mean (SE) of measures of glucose homeostasis and insulin resistance and secretion in men across categories of birth weight, stratified by maternal diabetes status: ELSA-Brasil (2008–2010).

Birth weight categories				
	<2.5 kg Mean (SE)	≥2.5, ≤4 kg Mean (SE)	>4 kg Mean (SE)	*P*-value
**Men whose mothers had history of diabetes**^**1**^				
**Fasting glucose (mmol/l)**				
Model 1	6.16 (0.13)	6.10 (0.07)	6.16 (0.10)	0.78
Model 2	6.19 (0.13)	6.10 (0.07)	6.11 (0.10)	0.75
**2 h postload glucose (mmol/l)**				
Model 1	7.41 (0.32)	7.60 (0.18)	7.50 (0.25)	0.77
Model 2	7.54 (0.31)	7.59 (0.17)	7.30 (0.24)	0.42
**HOMA-IR**				
Model 1	1.79 (0.23)	1.79 (0.12)	1.98 (0.20)	0.55
Model 2	1.98 (0.21)	1.78 (0.10)	1.69 (0.14)	0.41
**HOMA-β**^**†**^				
Model 1	51.18 (3.04)	53.04 (1.70)	52.24 (2.44)	0.78
Model 2	51.27 (3.05)	53.03 (1.70)	52.12 (2.45)	0.78
**ISI-composite**				
Model 1	4.34 (0.53)	4.43 (0.29)	4.26 (0.41)	0.90
Model 2	3.95 (0.40)	4.45 (0.25)	4.95 (0.40)	0.13
**Men whose mothers did not have history of diabetes**^**2**^				
**Fasting glucose (mmol/l)**				
Model 1	6.10 (0.05)	6.11 (0.03)	6.08 (0.05)	0.82
Model 2	6.11 (0.05)	6.10 (0.03)	6.02 (0.05)	0.17
**2 h postload glucose (mmol/l)**				
Model 1	7.26 (0.14)	7.32 (0.08)	7.13 (0.13)	0.30
Model 2	7.29 (0.13)	7.29 (0.08)	6.92 (0.12)	0.005
**HOMA-IR**				
Model 1	1.48 (0.09)	1.68 (0.06)	1.84 (0.10)	0.008
Model 2	1.52 (0.08)	1.65 (0.05)	1.58 (0.07)	0.13
**HOMA-β**^**†**^				
Model 1	46.02 (1.11)	46.80 (0.64)	47.94 (1.09)	0.34
Model 2	46.02 (1.11)	46.83 (0.63)	48.38 (1.10)	0.18
**ISI-composite**				
Model 1	5.28 (0.29)	4.90 (0.15)	4.82 (0.25)	0.29
Model 2	5.18 (0.25)	5.00 (0.13)	5.57 (0.25)	0.02

Model 1: adjusted for age, study center, race/color, maternal education, father diagnosed with diabetes; Model 2: + BMI at baseline.

^†^Further adjusted for HOMA-IR. *P*-value represents the test for an overall association of the different categories of estimated birth weight with each respective outcome.

^1^N = 914.

^2^N = 4076.
