# Supplementary material for: Sex-specific associations of low birth weight with adult-onset diabetes and measures of glucose homeostasis: Brazilian Longitudinal Study of Adult Health
Source: Sci Rep. 2016 Nov 15;6:37032. doi: 10.1038/srep37032 (PMC5109479; doi:10.1038/srep37032)
Supplement: Supplementary Information [file srep37032-s1.pdf]

Sex-specific associations of low birth weight with adult-onset diabetes and measures of glucose homeostasis: Brazilian Longitudinal Study of Adult Health

James Yarmolinsky, Noel T Mueller, Bruce B Duncan, Dóra Chor, Isabela M Bensenor, Rosane H Griep, Lawrence J Appel, Sandhi M Barreto, Maria Inês Schmidt

Supplemental Table S1. Multivariable adjusted prevalence ratios (and 95% confidence intervals) for the association of birth weight categories with adult-onset diabetes, stratified by sex and maternal diabetes, among participants with a precise birth weight estimate: ELSA-Brasil

	Birth weight categories			
	< 2.5 kg	≥ 2.5, ≤ 4 kg	> 4 kg	*P*-value
**Men**				
n	433	2308	425	
Cases	114	459	82	
Model 1	1.20 (1.00-1.43)	1 (reference)	.97 (.79-1.19)	.15
Model 2	1.06 (.89-1.27)	1 (reference)	.92 (.75-1.12)	.50
Model 3†	1.12 (.93-1.33)	1 (reference)	.83 (.68-1.02)	.06
**Women**				
n	580	2841	306	
Cases	136	376	45	
Model 1	1.64 (1.38-1.95)	1 (reference)	1.06 (.81-1.40)	<.0001
Model 2	1.54 (1.29-1.82)	1 (reference)	1.01 (.77-1.33)	<.0001
Model 3†	1.54 (1.30-1.82)	1 (reference)	.88 (.68-1.15)	<.0001
**Maternal diabetes**				
n	213	967	200	
Cases	91	213	47	
Model 1	1.76 (1.45-2.13)	1 (reference)	.96 (.73-1.26)	<.0001
Model 2‡	1.74 (1.42-2.12)	1 (reference)	.96 (.74-1.26)	<.0001
Model 3	1.75 (1.44-2.14)	1 (reference)	.86 (.66-1.13)	<.0001
**No maternal diabetes**				
n	800	4182	531	
Cases	159	622	80	
Model 1	1.22 (1.05-1.43)	1 (reference)	.97 (.79-1.19)	.05
Model 2‡	1.11 (.95-1.30)	1 (reference)	.98 (.80-1.20)	.41
Model 3	1.15 (.98-1.34)	1 (reference)	.85 (.69-1.05)	.05

Model 1: adjusted for age, study center; Model 2: + race/color, maternal education, father diagnosed with diabetes; Model 3: + BMI at baseline. † Further adjusted for mother diagnosed with diabetes. ‡ Further adjusted for sex. *P*-value represents the test for an overall association of the different categories of estimated birth weight with diabetes

Supplemental Table S2. Multivariable adjusted prevalence ratios (and 95% confidence intervals) for the association of birth weight categories with adult-onset diabetes, stratified by sex and maternal diabetes, excluding pre-term births: ELSA-Brasil

	Birth weight categories			
	< 2.5 kg	≥ 2.5, ≤ 4 kg	> 4 kg	*P*-value
**Men**				
n	302	4537	535	
Cases	79	1012	104	
Model 1	1.14 (.94-1.38)	1 (reference)	.91 (.77-1.09)	.22
Model 2	1.07 (.89-1.29)	1 (reference)	.89 (.75-1.06)	.30
Model 3†	1.10 (.91-1.34)	1 (reference)	.81 (.68-.96)	.02
**Women**				
n	374	5740	395	
Cases	96	846	56	
Model 1	1.73 (1.45-2.07)	1 (reference)	.99 (.78-1.26)	<.0001
Model 2	1.62 (1.35-1.92)	1 (reference)	.96 (.76-1.22)	<.0001
Model 3†	1.60 (1.34-1.90)	1 (reference)	.82 (.65-1.03)	<.0001
**Maternal diabetes**				
n	148	1995	250	
Cases	60	498	57	
Model 1	1.59 (1.30-1.94)	1 (reference)	.88 (.70-1.12)	.0006
Model 2‡	1.55 (1.26-1.90)	1 (reference)	.90 (.71-1.14)	.002
Model 3	1.54 (1.25-1.89)	1 (reference)	.82 (.65-1.03)	.0004
**No maternal diabetes**				
n	528	8282	680	
Cases	115	1360	103	
Model 1	1.30 (1.10-1.53)	1 (reference)	.92 (.77-1.10)	.01
Model 2‡	1.21 (1.03-1.43)	1 (reference)	.93 (.78-1.11)	.07
Model 3	1.24 (1.04-1.47)	1 (reference)	.81 (.68-.97)	.002

Model 1: adjusted for age, study center; Model 2: + race/color, maternal education, father diagnosed with diabetes; Model 3: + BMI at baseline. † Further adjusted for mother diagnosed with diabetes. ‡ Further adjusted for sex. *P*-value represents the test for an overall association of the different categories of estimated birth weight with diabetes
